# Engagement analysis of a persuasive-design-optimized eHealth intervention through machine learning

**DOI:** 10.1038/s41598-024-72162-z

**Published:** 2024-09-13

**Authors:** Abdul Rahman Idrees, Felix Beierle, Agnes Mutter, Robin Kraft, Patricia Garatva, Harald Baumeister, Manfred Reichert, Rüdiger Pryss

**Affiliations:** 1 Institute of Databases and Information Systems, 89081 Ulm, Germany; 2 Department of Clinical Psychology and Psychotherapy, 89081 Ulm, Germany; 3 Institute of Clinical Epidemiology and Biometry, 97070 Würzburg, Germany; 4 National Institute of Informatics, Tokyo, 101-8430 Japan; 5 Institute of Medical Data Science, University Hospital Würzburg, 97080 Würzburg, Germany

**Keywords:** Digital interventions, eHealth, Machine learning, Persuasive design, User engagement, Human behaviour, Randomized controlled trials, Computer science, Data processing

## Abstract

The challenge of sustaining user engagement in eHealth interventions is a pressing issue with significant implications for the effectiveness of these digital health tools. This study investigates user engagement in a cognitive-behavioral therapy-based eHealth intervention for procrastination, using a dataset from a randomized controlled trial of 233 university students. Various machine learning models, including Decision Tree, Gradient Boosting, Logistic Regression, Random Forest, and Support Vector Machines, were employed to predict patterns of user engagement. The study adopted a two-phase analytical approach. In the first phase, all features of the dataset were included, revealing ‘total_minutes’—the total time participants spent on the intervention and the eHealth platform—as the most significant predictor of engagement. This finding emphasizes the intuitive notion that early time spent on the platform and the intervention is a strong indicator of later user engagement. However, to gain a deeper understanding of engagement beyond this predominant metric, the second phase of the analysis excluded ‘total_minutes’. This approach allowed for the exploration of the roles and interdependencies of other engagement indicators, such as ‘number_intervention_answersheets’—the number of completed lessons, ‘logins_first_4_weeks’—login frequency, and ‘number_diary_answersheets’—the number of completed diaries. The results from this phase highlighted the multifaceted nature of engagement, showing that while ‘total_minutes’ is strongly correlated with engagement, indicating that more engaged participants tend to spend more time on the intervention, the comprehensive engagement profile also depends on additional aspects like lesson completions and frequency of platform interactions.

## Introduction

The high prevalence of procrastination among university students has become a cause for concern, as it is linked to lower academic performance, higher stress levels, and reduced well-being^1,2^. In recent years, the use of eHealth applications, particularly those based on cognitive-behavioral therapy (CBT), has emerged as a promising approach to addressing the issue of procrastination^3,4^. However, the success rates of these eHealth interventions are often influenced by the level of user engagement with the platform^5,6^. In this context, the current study extends its analytical purview by evaluating various machine learning models, examining if the predictive capacity is model-specific or consistent across different approaches. In this work, we conducted a secondary analysis of a randomized controlled trial (RCT) that evaluated the non-inferiority of a digital coach compared to human guidance in reducing procrastination^7^. The intervention, based on CBT, was designed to help university students manage procrastination. Participants were divided into two groups: the intervention group (IG), which received guidance from a digital coach with a selectable avatar, and the control group (CG), which received guidance from a trained guide with a bachelor’s degree in psychology. Both groups were exposed to the same CBT-based intervention, optimized with persuasive design (PD) features including tunneling, social support, tailoring, personalization, and trustworthiness, with the primary difference being the guidance method. PD is an area of research that focuses on the strategic use of psychological and design principles to influence human behavior and decision-making toward a desired outcome. According to Fogg^8^, PD involves utilizing computer technologies to alter attitudes or behaviors without resorting to force or trickery. PD can incorporate various strategies, such as tunneling, where the system guides users through a sequence of steps, allowing for persuasive elements to be introduced along the way; tailoring, which adapts information to match the user’s specific needs, interests, or context; personalization, where content or services are individualized to enhance persuasiveness; trustworthiness, which increases the system’s credibility and thus its persuasive effectiveness; suggestion, where timely recommendations are made to influence user behavior; and reduction, which simplifies complex behaviors into more manageable actions, making it easier for users to achieve their goals^9^. While several studies have demonstrated the potential of PD in eHealth applications^10–12^, the comparative effectiveness of these design features when combined with different guidance methods, such as a digital coach or a human coach, has received limited attention.

The primary objective of this secondary analysis was to examine participant engagement with the intervention and its eHealth platform, emphasizing key engagement measures. These measures include metrics such as the number of logins, time spent on the platform, and completion of certain activities. Additionally, the study explores engagement patterns with the intervention and the eHealth platform, particularly how variables, such as age, gender, country of origin, therapy status, and frequency of use, influence these patterns. Detailed definitions of these measures and variables are provided in the methods section. In particular, this research aims to address the following question:

*Is it possible to predict which participants are more likely to disengage from the intervention prematurely (e.g., drop out or complete fewer lessons) based on early engagement data?*

The paper is organized as follows: the Methods section describes the methodology used in the RCT and the secondary analysis; the Results section presents the findings of this analysis; the Discussion section discusses the implications of these findings; the Limitations section highlights the limitations of this work; and finally, the Conclusion section concludes the paper.

## Methods

### Study design

This secondary analysis is primarily aimed at examining participant engagement with the procrastination intervention and its associated eHealth platform, with a particular emphasis on key engagement measures. The focus is on the patterns of engagement, investigating how various factors, including demographics, affect these patterns within the context of a previously conducted RCT^7^. To comprehensively understand engagement dynamics, assessments were conducted at multiple stages: baseline (t0), four weeks (t1), eight weeks (t2), 12 weeks (t3), six months (t4), and 12 months (t5) of the RCT. Data collection began in June 2021 and concluded in August 2022. The majority of the data was gathered between June 2021 and January 2022, though some participants completed the intervention over an extended period, leading to an ongoing data collection process for several months. During the data collection period, no widespread external factors, such as COVID-19 lockdowns, uniformly impacted all participants. The staggered recruitment and participation timelines may have led to some variability in data collection timing across individuals.

### Intervention

The intervention aimed to assist students in managing their procrastination tendencies. The intervention consisted of six lessons, with the first lesson serving as an introduction and an optional lesson following the fifth one. The lessons were offered in German and were released sequentially, with one lesson made available each week. However, participants had the flexibility to complete the lessons without any specific deadlines. Each lesson included a weekly challenge, and participants could choose to track their progress using the diary feature.

Each module in the intervention was structured using a variety of formats, including written texts, videos, audio recordings, images, and interactive elements, to facilitate effective learning and application. The first module focused on educating participants about procrastination, outlining its causes and symptoms, and introducing the Rubicon Model of Action Phases^13^. This module also incorporated exercises for self-monitoring. The second module concentrated on developing time management skills, covering topics such as goal setting, task prioritization, and planning. The third module addressed motivational techniques, allowing participants to examine their individual motivation patterns. The fourth module provided strategies for self-regulation and self-control and included mindfulness exercises aimed at relaxation. The fifth module reviewed all the theories and strategies presented in the previous modules, included reflective exercises, and offered guidance on managing relapses. An optional sixth module was available, which addressed issues related to self-worth, perfectionism, and fear of failure, and provided exercises to assist participants in managing these challenges. For additional information regarding the intervention, refer to^14^.

When working through the intervention, participants generate two distinct types of answer sheets: intervention and diary answer sheets. A new intervention answer sheet is created upon the completion of each lesson. Additionally, every instance of engaging with their daily diary prompts the creation of a new diary answer sheet.

To enhance participant adherence and engagement, the intervention was optimized using PD techniques as outlined in^9^. The intervention included several PD strategies. A personalization strategy was implemented by incorporating the participant’s name throughout the app. Self-monitoring was facilitated through the use of progress bars and daily diaries, assisting participants in tracking their progress in each lesson. To increase the intervention’s credibility, the study team was introduced to the users, accompanied by the provision of evidence-based materials. A tunneling strategy was applied by releasing the lessons to participants sequentially. This step-by-step approach was designed to engage participants more effectively with each piece of content, reinforcing the learning objectives of the current lesson before progressing to the next. It also aimed to prevent information overload and maintain the participants’ focus throughout the intervention. Furthermore, to provide social support, participants were paired in teams of two with other participants from their respective groups, aiming to offer motivational feedback and reminders for lesson completion. The feedback and reminder content, which was predefined and unchangeable, was exchanged between participants via email.

### eSano platform

The eSano eHealth platform was used to deliver the intervention. This platform was designed to be adaptable, allowing for the creation, customization and delivery of new interventions as required. eSano is an eHealth platform for Internet- and mobile-based interventions and is composed of three sub-platforms: a Content Management System (CMS), an eCoach platform, and a Participant app. The Content Management System (CMS) is a tool that helps researchers develop interventions without any background in IT. The eCoach platform enables the supervision of individuals who sign up for an intervention. The Participant app is a cross-platform application that allows individuals to access their interventions through any internet-connected device, including desktop computers, smartphones, and tablets. The content of the interventions on eSano is delivered through text-based means as well as multimedia formats, such as images and videos. Furthermore, various interactive tools are available to help participants engage with the content and provide responses to the different lessons of an intervention. Study participants accessed the intervention via the participant app of the platform. Additional details regarding the platform are available in^15^, which describes the platform, its requirements, and its architecture. Moreover^16^, provides more details about eSano with a focus on its backend.

### Recruitment and randomization

Recruitment for the RCT was conducted between May and September 2021. Participants were enrolled on a staggered basis, meaning they did not all begin the intervention simultaneously. Participants were recruited through multiple channels. These channels included the trial management system of Ulm University, cooperating universities in Germany, Austria, and Switzerland, in addition to flyers and posters. Participants were recruited on a voluntary basis. No incentives, financial or otherwise, were provided to engage in the study or the intervention. To be eligible for participation, candidates were required to be at least 18 years old, proficient in the German language, have access to an Internet-connected device such as a smartphone or a notebook, and be registered as students. Candidates who met these criteria were assessed for a procrastination level of 32 or higher on the Irrational Procrastination Scale (IPS $\ge$ 32)^17^. Participants were randomized by a researcher not otherwise involved in the trial in a 1:1 ratio between the two arms.

### Guidance

In the IG, participants received guidance from a digital coach. They could choose the avatar of their coach, which could represent either a male or a female. The coach provided feedback based on each participant’s input. For example, if a participant completed a lesson, they received motivational feedback. If they stopped using the intervention, a reminder email was sent after a pre-selected amount of time. In this case, participants received a reminder email 12 days after their self-selected date of intervention continuation. Additionally, participants received a standardized module summary and motivational feedback two days after completing each module. At the end of each module, participants scheduled their next module appointment. If they missed this appointment, they received a reminder email 12 days later. Throughout each module, the digital coach provided immediate, standardized feedback based on the participant’s responses. In contrast, the CG received human guidance from a trained guide with at least a bachelor’s degree in psychology. Feedback was given only after completing each lesson, and the next lesson became available after reading the feedback. This feedback was a mix of standardized content and personalized text based on the participants’ entries. In addition, CG participants received reminders three, seven, ten, and twelve days after the start of each lesson.

### Ethics approval

The methods used in this trial were approved by the Ethics Committee of Ulm University (Ulm, Germany; 502/20) and preregistered at the German Clinical Trials Register (DRKS00025209). The trial was conducted according to “the CONSORT Guidelines for Noninferiority and Equivalence Trials Studies”^18^. Detailed informed consent was obtained from all participants to ensure that they understood the procedures of the study and their rights, including the option to withdraw at any time without any consequences. No personally identifiable information or images were included in this paper.

### Demographics

At the start of the study, we gathered demographic information about participants, including age, gender, field of study, state, number of completed semesters, current exam preparation, semester break, and experience with psychotherapy.

### Data analysis

To answer our research question, we employed the following data analysis methods using Python (version 3.9) in an Anaconda environment with key libraries including *pandas*, *numpy*, *optuna*, *matplotlib*, *scikit-learn*, *imbalanced-learn*, and SHapley Additive exPlanations^19^ *(SHAP)* (https://shap.readthedocs.io/en/latest).

#### Target variable

In this study, the ’late_engagement’ variable is designed to describe participant engagement in an eight-week intervention, specifically focusing on engagement from week five onwards due to observed decreases in usage during the later weeks. Engagement status, classified as ’engaged’ (1) or ’disengaged’ (0), is predicted based on a variety of features from the first four weeks of participant interaction. These features include interaction metrics like the number of logins, the number of submitted intervention answer sheets, the number of submitted diary answer sheets, and social support interactions. Participants were classified as “engaged” based on a holistic assessment of their interaction with the platform. Specifically:

- A participant who logged in multiple times but did not complete any new lessons or daily tasks was not classified as engaged, as mere logins without substantive interaction do not reflect meaningful engagement.
- Similarly, participants who spent significant time on the platform but failed to complete any lessons were also considered disengaged, as time spent without achieving key milestones indicates a lack of active participation.
- Conversely, participants who completed several lessons but spent only minimal time on each were not considered engaged either. This behavior suggests a focus on quickly progressing through the content rather than a thorough engagement with the material, which typically requires adequate time to absorb and reflect on the lessons.
- Participants who demonstrated a balanced interaction by completing lessons, spending a sufficient amount of time on each, engaging with daily tasks, and actively participating in social support interactions were classified as engaged.

This classification approach was developed in consultation with experts in the field, thereby aligning it with realistic expectations of participant behavior and the objectives of the intervention. Additionally, it helps identify meaningful engagement that contributes to the intervention’s efficacy, rather than merely superficial interaction with the platform. Additional features encompass demographic information (age, gender, state), study-related variables (study subject, semester break), therapy status (current, past, waiting list, none), and scores from the Irrational Procrastination Scale (IPS) at baseline and after four weeks.

#### Features

The dataset included a diverse array of features, broadly categorized into demographic data, therapy status, and interaction metrics:

1. Demographic Data: This included age, gender, country of residence, and the study subject/major, with all participants being students.
2. Therapy Status: The dataset captured various aspects of therapy engagement:
  current_therapy: Indicates ongoing therapy participation.
  past_therapy: Denotes previous therapy experience without current engagement.
  waiting_therapy: Reflects an anticipation or plan to commence therapy.
3. Interaction Features: These features provided insights into the participants’ interaction with the intervention platform:
  Number of logins (logins_first_4_weeks): The total number of times a participant logged into the platform during the first four weeks.
  Total time spent on the platform (total_minutes): The cumulative time spent by participants on the platform.
  Number of intervention answer sheets (number_intervention_answersheets): The number of intervention lessons completed by a participant during the first four weeks.
  Number of social support activities (number_sent_reminders and number_times_adherence_reminders): The number of social support-related activities a participant engaged in.
  Number of completed daily diaries (number_diary_answersheets): The total number of daily diaries completed by participants.
  First login’s day of the week (first_login_day_of_week): The day of the week on which the participant first logged into the platform.
  Age band categorization (age_band): The categorization of participants into different age bands.
  Interaction of age band and gender (age_gender_interaction): A composite feature combining age band and gender to study their joint effect on intervention interaction.

In total, 13 features were considered in the analysis, categorized as follows: 4 features in Demographic Data, 1 feature in Therapy Status, and 8 features related to user interaction with the intervention.

#### Feature engineering

To enhance the dataset’s analytical utility, several derived features were formulated:

- Age band: This categorical feature was extrapolated from the continuous ‘age’ variable. Age groups were systematically categorized (e.g., ‘18–24’, ‘25–30’, etc.), aiding in the analysis by simplifying age-related trends.
- Age band and gender interaction: This composite feature integrates age bands with gender, creating a unique identifier for each combination. It aims to capture the nuanced interplay between age and gender in participant engagement.
- Therapy status: The nuanced therapy status of participants was encapsulated through “current_therapy”, “past_therapy”, and “waiting_therapy”. These features were meticulously derived to distinguish between different phases of therapy engagement, enriching the dataset with layers of behavioral insight.
- First login’s day of the week: This feature captures the day of the first login of each participant.

Initial analysis showed that ‘total_minutes’ was a significantly dominant predictor. To understand the impact of other variables on engagement, a subsequent analysis was performed excluding ‘total_minutes’. This exclusion serves as an ablation study, aimed at uncovering the relative importance of other features and providing a more comprehensive understanding of user interaction beyond the primary metric of time spent on the application.

#### Machine learning models

Following the classification of participants as engaged or disengaged, we began the exploration of machine learning models to forecast user engagement. We employed several models, namely Logistic Regression, Decision Trees, Random Forests, Support Vector Machines (SVM), and Gradient Boosting. This choice was motivated by their wide use in the field of machine learning for binary classification problems such as ours. Alongside this, an additional aim was to investigate whether the selection of the model itself significantly impacts the prediction of user engagement. This approach allowed for a side-by-side comparison to identify if some models provided more accurate or consistent predictions in the context of this study. For each model, the following steps were performed:

1. *Data preprocessing:* The dataset consisted of a mixture of numerical and categorical variables. Numerical variables were standardized using StandardScaler, while categorical variables underwent one-hot encoding with OneHotEncoder, to normalize their range and format, respectively.
2. *Data splitting:* a nested cross-validation approach was employed using StratifiedKFold with 5 splits, to ensure a balanced representation of classes in each fold. This method divided the data into separate training and testing sets in each iteration.
3. *Oversampling:* Due to imbalanced class distribution, SMOTE^20^ was applied to generate synthetic samples for the minority class in the training data.
4. *Model training:* Each machine learning model was trained using hyperparameters optimized with Optuna (an Open-Source framework designed for automatic hyperparameter optimization^21^), fine-tuned to their specific requirements:
  1. *Decision tree:* The hyperparameters max_depth (ranging from 1 to 40), min_samples_split (between 2 and 10), and min_samples_leaf (from 1 to 10) were adjusted.
  2. *Gradient boosting:* Adjustments included ‘n_estimators‘ (50 to 200), ‘learning_rate‘ (0.01 to 0.2), ‘max_depth‘ (1 to 7), ‘min_samples_split‘ (2 to 10), ‘min_samples_leaf‘ (1 to 10), and ‘subsample‘ (0.5 to 1).
  3. *Logistic regression:* Adjustments were conducted for the regularization parameter ‘C‘ (1e-4 to 1e4) and the ‘penalty‘ type (either ‘l1’ or ‘l2’), with the solver selection corresponding to the penalty type.
  4. *Random forests:* Adjustments included ‘n_estimators‘ (2 to 150), ‘max_depth‘ (1 to 40), ‘min_samples_split‘ (2 to 10), and ‘min_samples_leaf‘ (1 to 10).
  5. *Support vector machine (SVM):* The adjustment process included ‘C‘ (1e-4 to 1e4), ‘kernel‘ type (options: ‘linear’, ‘poly’, ‘rbf’, ‘sigmoid’), ‘degree‘ (1 to 5 for ‘poly’ kernel), ‘gamma‘ (‘scale’ or ‘auto’), and ‘coef0‘ (− 1.0 to 1.0).
5. *Model evaluation:* The performance of the models was assessed through accuracy and Precision-Recall Area Under Curve (PR-AUC) metrics. The PR-AUC provides a reliable method for assessing the models in the face of class imbalance. This metric is particularly important as it balances the focus between precision (the model’s ability to identify true positives) and recall (the model’s ability to capture all actual positives), which is often more informative than just accuracy in imbalanced datasets. SHAP values were also computed to interpret the model’s decision-making process, offering insights into feature importance. To compare the performance between the first and second iterations of the models, the percentage difference in accuracy and PR-AUC was calculated. The percentage difference was determined using the following formula:
  1 $$\begin{aligned} \text {Diff (\%)} = \frac{\text {Value (1st)} - \text {Value (2nd)}}{\text {Value (1st)}} \times 100 \end{aligned}$$
  This formula expresses the relative change between the first and second iterations, allowing for a standardized comparison of the model performance.
6. *Statistical analysis:* Accuracy, classification reports, and confusion matrices were computed for each cross-validation fold to evaluate the performance of the models. A cumulative Precision-Recall Curve was plotted to visualize the trade-off between precision and recall across different thresholds.

All images presented in this paper were generated by the authors as part of this data analysis and are not reproductions from other sources.

## Results

This section presents the results of the secondary analysis. Initially, it presents the results of the exploratory data analysis. Following this, it displays the outcomes of five machine learning models over two iterations. The first iteration utilizes all the available features of the dataset. The second iteration also uses all the features of the dataset, but excludes the most influential feature identified in the first iteration.

### Exploratory data analysis (EDA)

**Figure 1:**
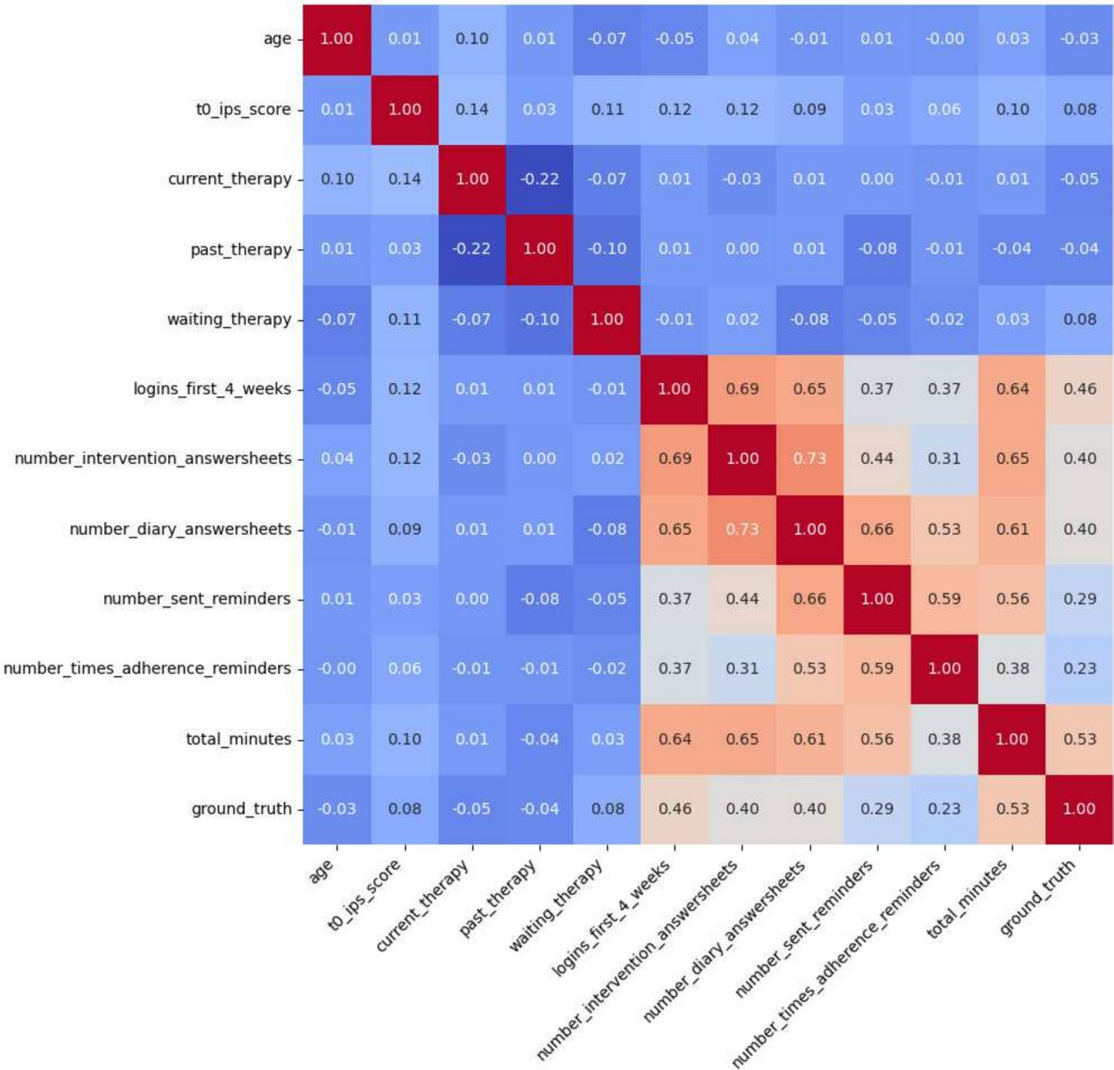
Spearman correlation matrix.

The dataset comprised 233 participants (140 female, 90 male). The demographic and engagement status distribution of participants is summarized in Table 1, while Table 2 presents interaction metrics. The Spearman correlation matrix is visualized in Fig. 1. It can be seen that ‘logins_first_4_weeks’, ‘number_diary_answersheets’, and ‘total_minutes’ demonstrated significant associations with the outcome variable ‘late_engagement’. The ‘logins_first_4_weeks’ had the strongest correlation (r = 0.370, $p<$ .001), followed by ‘number_diary_answersheets’ (r = 0.338, $p<$ .001), and ‘total_minutes’ (r = 0.336, $p<$ .001).

The Point Biserial Correlation analysis between ‘late_engagement’ and continuous variables indicated the highest correlations with ‘logins_first_4_weeks’ (r = .370, $p<$ .001), ‘number_diary_answersheets’ (r = .338, $p<$ .001), and ‘total_minutes’ (r = .336, $p<$ .001) as can be seen in Table 3. Other variables, including ‘age’, ‘t0_ips_score’, ‘current_therapy’, ‘past_therapy’, and ‘waiting_therapy’, showed no significant correlations with ‘late_engagement’ ($p>$ .05).

The dataset exhibited a small proportion of missing data, specifically within the ‘first_login_day_of_week’ variable, for which 19 entries were not recorded. However, these missing values were not due to data entry errors or loss during data collection, but were characteristic of the study’s design. The absence of data in this variable indicates participants who did not engage with the intervention during the study period. This non-engagement is still considered informative.

**Table 1:** Demographic and engagement status distribution of participants.

Variable	Description
Age range:	19–53; Mean (M) = 26.21; SD* = 5.35
Gender	Female: 60.1% (140), Male 38.6% (90), Other: 1.3% (3)
Disengaged	58.4% (Total number: 136)
Engaged	41.6% (Total number: 97)

*SD = Standard Deviation

**Table 2:** Interaction metrics.

Metric	Min	Max	M*	SD*
logins_first_4_weeks	0	37	6.73	6.04
number_intervention_answersheets	0	11	2.76	2.13
number_diary_answersheets	0	25	3.25	5.26
number_sent_reminders	0	16	1.28	2.61
number_times_adherence_reminders	0	7	0.38	1.15
total_minutes	0	571.4	57.1	80.6

*M = Mean, SD = Standard Deviation

**Table 3:** Point biserial correlation coefficients for key features with late engagement.

Feature	Correlation (r)	*P*-value
logins_first_4_weeks	0.370	< .001
number_intervention_answersheets	0.327	< .001
number_diary_answersheets	0.338	< .001
number_sent_reminders	0.249	< .001
number_times_adherence_reminders	0.273	< .001
total_minutes	0.336	< .001

### First Iteration

The first iteration of results reveals the performance metrics of the five machine learning models, as summarized in Tables 4 and 5. The Decision Tree model in the first iteration demonstrated precision values ranging from 0.75 to 0.86 for Class 0 and 0.68 to 0.93 for Class 1, while recall varied from 0.78 to 0.96 for Class 0 and 0.65 to 0.79 for Class 1. Gradient Boosting’s precision for Class 0 hovered around 0.74 to 0.88. Logistic Regression exhibited precision between 0.68 and 0.91 for Class 0. Random Forest showed precision from 0.78 to 0.87 for Class 0, and SVM’s precision varied from 0.72 to 0.89 for the same class. In terms of SHAP values, ‘total_minutes’ emerged as a significant feature, especially in the Decision Tree and Gradient Boosting models, with mean SHAP values of 0.044 and 0.121 respectively. Other influential features included ‘logins_first_4_weeks’ and ‘number_diary_answersheets’, with notable mean SHAP values in different models.

**Table 4:** Range of performance metrics for different models—1st iteration.

Model	Precision		Recall		F1-Score		Accuracy	PR-AUC
	0	1	0	1	0	1		
Decision tree	[0.75, 0.86]	[0.68, 0.93]	[0.78, 0.96]	[0.65, 0.79]	[0.76, 0.88]	[0.67, 0.81]	[0.72, 0.85]	[0.74, 0.86]
Gradient boosting	[0.74, 0.88]	[0.65, 0.88]	[0.74, 0.93]	[0.65, 0.84]	[0.74, 0.88]	[0.65, 0.81]	[0.70, 0.85]	[0.63, 0.88]
Logistic regression	[0.68, 0.91]	[0.64, 0.78]	[0.70, 0.93]	[0.37, 0.89]	[0.73, 0.84]	[0.50, 0.77]	[0.70, 0.80]	[0.57, 0.76]
Random forests	[0.78, 0.87]	[0.62, 0.87]	[0.67, 0.93]	[0.65, 0.84]	[0.72, 0.85]	[0.68, 0.75]	[0.70, 0.81]	[0.61, 0.82]
SVM	[0.72, 0.89]	[0.59, 0.72]	[0.57, 0.85]	[0.53, 0.89]	[0.70, 0.79]	[0.61, 0.74]	[0.68, 0.74]	[0.56, 0.78]

**Table 5:** Average of performance metrics for different models—1st Iteration.

Model	Precision		Recall		F1-Score		Accuracy	PR-AUC
	0	1	0	1	0	1		
Decision tree	0.81	0.78	0.84	0.72	0.83	0.75	0.79	0.81
Gradient boosting	0.83	0.74	0.80	0.76	0.81	0.75	0.79	0.78
Logistic regression	0.77	0.72	0.81	0.64	0.78	0.66	0.74	0.67
Random forests	0.83	0.71	0.76	0.77	0.79	0.73	0.76	0.72
SVM	0.80	0.65	0.71	0.73	0.74	0.68	0.72	0.67

**Figure 2:**
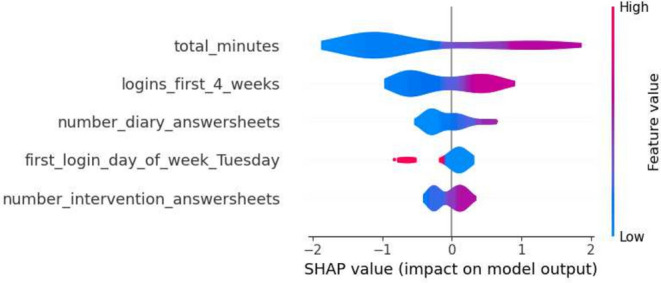
Gradient boosting fold 1 SHAP plot—1st iteration.

**Figure 3:**
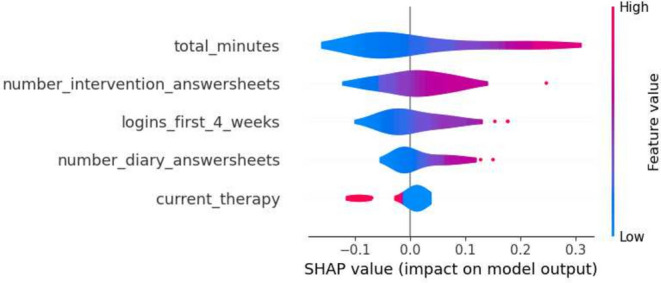
SVM fold 2 SHAP plot—1st iteration.

**Figure 4:**
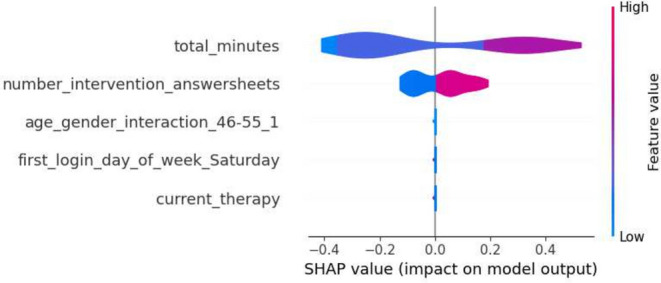
Decision tree fold 3 SHAP plot—1st iteration.

**Figure 5:**
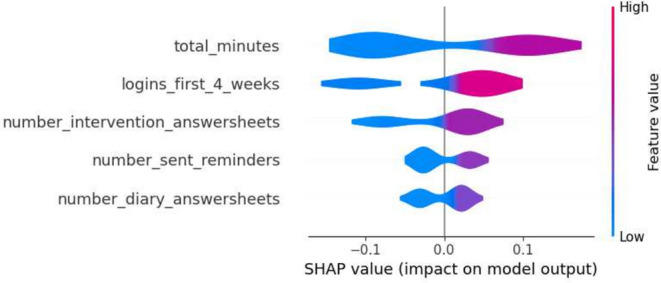
Random forest fold 4 SHAP plot—1st iteration.

**Figure 6:**
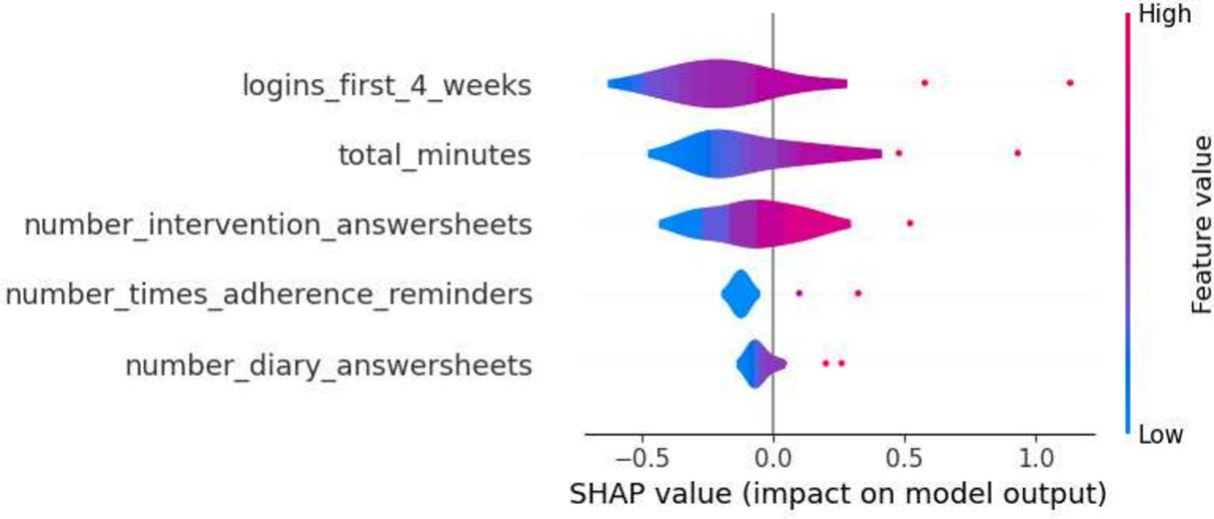
Logistic regression fold 5 SHAP plot—1st iteration.

Furthermore, a selection of SHAP plots for the machine learning models is presented in Figs. 2, 3, 4, 5 and 6 , offering a visual representation of feature influence on model outcome.

### Second Iteration

**Figure 7:**
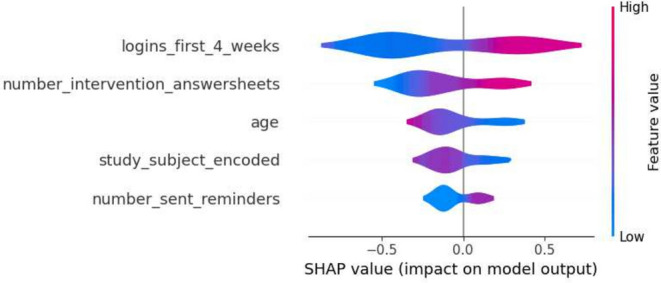
Gradient boosting fold 1 SHAP plot—2nd iteration.

**Figure 8:**
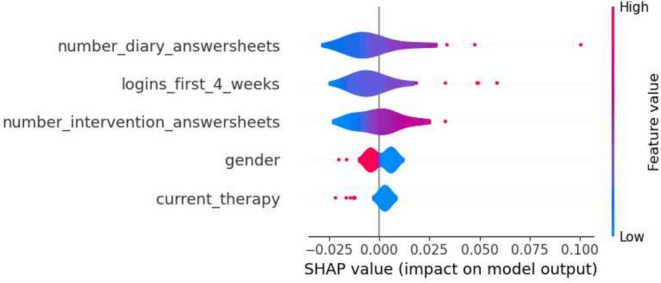
SVM fold 5 SHAP plot—2nd iteration.

**Fig. 9:**
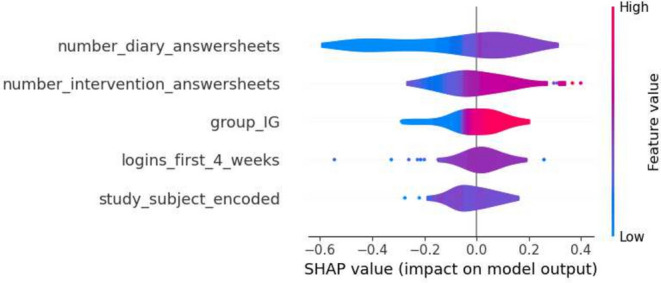
Decision tree fold 3 SHAP plot—2nd iteration.

This section details the results of the second iteration of the five machine learning models, conducted without the ‘total_minutes’ feature, which was identified as the most influential one in the first iteration. The performance metrics of each model are summarized in the tables in Tables 6 and 7. The Decision Tree’s precision for Class 0 in the second iteration ranged from 0.74 to 0.88, and for Class 1, it was between 0.25 and 0.58. Gradient Boosting showed precision between 0.77 and 0.86 for Class 0, and between 0.33 and 0.60 for Class 1. Logistic Regression’s precision for Class 0 was between 0.77 and 0.89, and for Class 1, it ranged from 0.36 to 0.64. The Random Forest model had precision values ranging from 0.77 to 0.84 for Class 0, and from 0.33 to 0.56 for Class 1. SVM’s precision varied from 0.77 to 0.86 for Class 0, and from 0.33 to 0.60 for Class 1. Moreover, the SHAP analysis highlighted ‘number_intervention_answersheets’ as a key feature, with its mean SHAP value in the Decision Tree model being 0.145. Other notable features included ‘number_diary_answersheets’ and ‘logins_first_4_weeks’, which showed significant mean SHAP values in various models. A selection of SHAP plots for the machine learning models is presented in Figs. 7, 8, 9 and 11 , offering a visual representation of feature influence on model outcome.

**Table 6:** Range of performance metrics for different models—2nd iteration.

Model	Precision		Recall		F1-Score		Accuracy	PR-AUC
	0	1	0	1	0	1		
Decision tree	[0.74, 0.88]	[0.25, 0.58]	[0.60, 0.86]	[0.17, 0.64]	[0.68, 0.87]	[0.20, 0.61]	[0.57, 0.80]	[0.27, 0.62]
Gradient boosting	[0.77, 0.86]	[0.33, 0.60]	[0.71, 0.89]	[0.25, 0.55]	[0.76, 0.87]	[0.30, 0.57]	[0.66, 0.80]	[0.28, 0.46]
Logistic regression	[0.77, 0.89]	[0.36, 0.64]	[0.74, 0.89]	[0.25, 0.67]	[0.78, 0.89]	[0.30, 0.64]	[0.67, 0.83]	[0.40, 0.59]
Random forests	[0.77, 0.84]	[0.33, 0.56]	[0.74, 0.89]	[0.33, 0.45]	[0.77, 0.86]	[0.33, 0.50]	[0.66, 0.78]	[0.31, 0.65]
SVM	[0.77, 0.86]	[0.33, 0.60]	[0.71, 0.89]	[0.25, 0.55]	[0.76, 0.87]	[0.30, 0.57]	[0.66, 0.80]	[0.28, 0.46]

**Table 7:** Average of performance metrics for different models—2nd iteration.

Model	Precision		Recall		F1-Score		Accuracy	PR-AUC
	0	1	0	1	0	1		
Decision tree	0.79	0.37	0.78	0.39	0.78	0.37	0.68	0.34
Gradient boosting	0.80	0.43	0.82	0.40	0.81	0.41	0.71	0.37
Logistic regression	0.83	0.47	0.81	0.50	0.82	0.48	0.73	0.49
Random forests	0.80	0.43	0.81	0.41	0.80	0.42	0.71	0.42
SVM	0.80	0.43	0.82	0.40	0.81	0.41	0.71	0.37

**Fig. 10:**
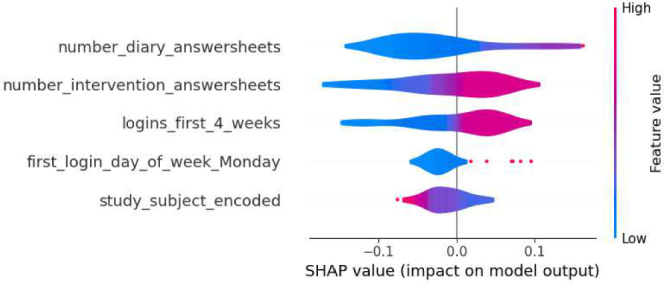
Random forest fold 4 SHAP plot—2nd iteration.

**Fig. 11:**
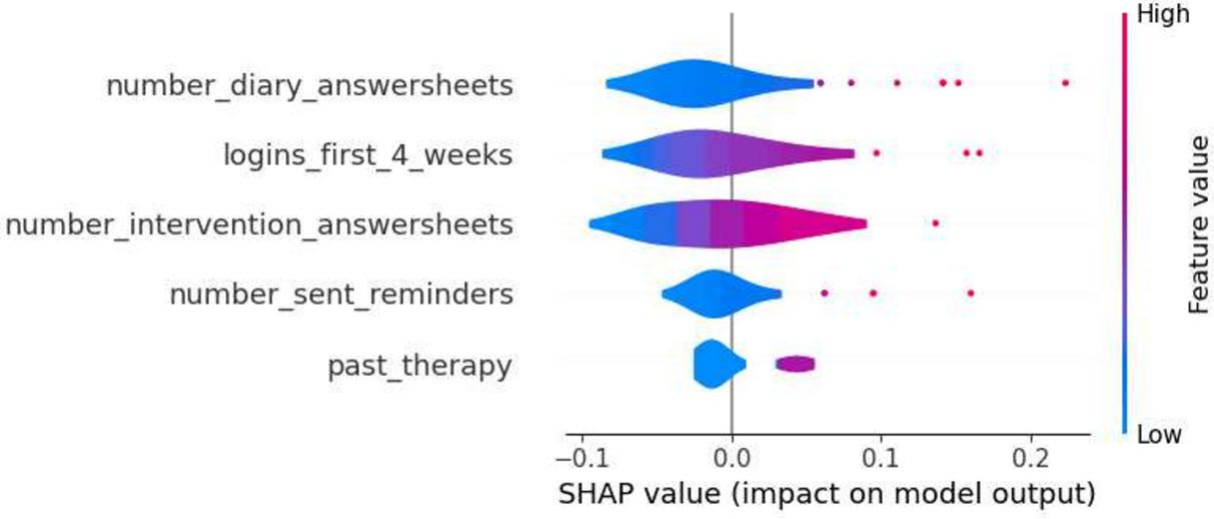
Logistic Regression fold 2 SHAP plot—2nd iteration.

**Table 8:** Comparison of accuracy and PR-AUC between first and second iterations for each model.

Model	Acc (1st)	Acc (2nd)	Acc Diff (%)	PR-AUC (1st)	PR-AUC (2nd)	PR-AUC Diff (%)
Decision tree	0.79	0.68	− 13.92%	0.81	0.34	− 58.02%
Gradient boosting	0.79	0.71	− 10.13%	0.78	0.37	− 52.56%
Logistic regression	0.74	0.73	− 1.35%	0.67	0.49	− 26.87%
Random forests	0.76	0.71	− 6.58%	0.72	0.42	− 41.67%
SVM	0.72	0.71	− 1.39%	0.67	0.37	− 44.78%

Table 8 summarizes the changes in accuracy and PR-AUC between the first and second iterations for each model. The table shows the accuracy (Acc) and PR-AUC for both iterations, along with the percentage change (Diff) between the two. The percentage change was calculated using Formula (1). The results indicate a noticeable decline in both accuracy and PR-AUC when the ‘total_minutes’ feature was excluded in the second iteration. For instance, the Decision Tree model experienced a 13.92% decrease in accuracy and a 58.02% decrease in PR-AUC. Similar trends were observed across other models, highlighting the significant role of ‘total_minutes’ in the models’ predictive performance.

## Discussion

The analytic exploration of predictive models in this study offers insights into user engagement within a digital intervention context. Throughout the two iterations of model evaluation, the feature ‘total_minutes’ stood out in the first iteration as a key determinant of user engagement. This supports the intuitive assumption that spending more time in the application is a strong indicator of user interest and engagement. However, the prominence of ‘total_minutes’ in the model’s decision-making process possibly overshadowed other meaningful dimensions of engagement. As defined in our study, engagement, extends beyond mere time spent in the platform, incorporating active participation elements such as lesson completions and frequency of logins.

The removal of ‘total_minutes’ in the second iteration, using an ablation study approach, highlighted the roles of ‘number_intervention_answersheets’, ‘logins_first_4_weeks’, and ‘number_diary_answersheets’ as significant engagement indicators. The correlation between increases in ‘total_minutes’ and higher engagement in intervention lessons and diary tasks, represented by these features, demonstrates the multi-dimensional nature of engagement, combining both time and depth of user interaction.

In the first iteration, the models exhibited varied PR-AUC scores, with Gradient Boosting and Decision Trees generally offering better performance. This suggests that these models were more adept at capturing the patterns associated with the ‘total_minutes’ feature, translating to a stronger ability to distinguish between engaged and disengaged participants. However, when comparing across the classifiers, no single model consistently outperformed the others in every metric. This variation in performance, even among the better-performing models, highlights the complexity of model selection in predicting user engagement, particularly in scenarios with imbalanced datasets.

The differences among the models became more evident when assessing their ability to capture complex, non-linear relationships and their response to changes in feature importance. With SHAP providing a consistent interpretability framework across models, the key differences were observed in how each model adapted to feature changes. Logistic Regression showed consistency and flexibility, performing steadily even when a major feature was removed. Gradient Boosting and Random Forests managed a balance between handling complexity and maintaining stable performance across a broader range of features. SVM, though less robust in some scenarios, displayed stable performance, making it a viable option under varying conditions. While correlations can certainly reveal important trends, this analysis suggests that machine learning models add value by capturing more complex patterns and relationships, particularly in how multiple features interact to predict engagement. For example, while the importance of total_minutes might have been identified through simpler analyses, the machine learning models revealed how its interaction with other features such as number_intervention_answersheets and logins_first_4_weeks contributed to engagement. This understanding of feature interactions and non-linear effects provides deeper insights that go beyond what correlations alone can reveal, enhancing the ability to predict engagement more accurately

The contrasting PR-AUC scores across iterations emphasize the importance of feature selection in model performance. The early weeks’ ‘total_minutes’ proved most predictive of later engagement, highlighting the impact of initial user involvement. This is a key finding for intervention and application developers, as it highlights the dual role of application design and user tendencies in shaping engagement.

Shifting the importance of the features from the first to the second iteration revealed insights into the interaction patterns of the participants. Focusing on various engagement indicators such as completed lessons and frequency of logging in allowed for a more nuanced view of participant behavior beyond just time spent on the platform. Consistent completion of lessons and regular logins, as opposed to a long period of time in a few sessions, indicate meaningful, sustained interaction, which is crucial for long-term retention.

This change in model performance suggests a need to reassess predictive features. While ‘total_minutes’ is a reliable predictor of future engagement, its dominant use in the models raises questions about the role of more granular features. The effectiveness of models using ‘total_minutes’ implies that early time spent is crucial for predicting future engagement. Nevertheless, this does not diminish the value of granular data, which offers a more detailed view of participant interactions with the intervention, essential for tailoring and enhancing user experience.

Furthermore, reinforcing the need for a nuanced understanding of engagement, the study by Chien et al.^22^ demonstrates that digital health interventions benefit from recognizing varied user engagement patterns. They found that different types of user interactions-ranging from goal-based activities to mood tracking-correlated with distinct clinical outcomes, highlighting the limitations of using ‘total_minutes’ alone as a measure of user engagement.

Moreover, the models’ performance suggests that behavioral patterns might be influenced by external factors not present in the dataset. These could include individual anxiety levels, social influences from peers or family, technology literacy, personal attitudes towards using technology, mood fluctuations, and personal preferences. Additionally, the insights from Borghouts et al.^23^ emphasize that personal experiences with technology, socio-economic barriers, and cultural expectations can significantly impact user engagement with digital health tools. This indicates the need to consider a broad spectrum of external factors in our models to better predict and enhance user engagement.

Recognizing such factors could provide a more comprehensive understanding of the models’ effectiveness and limitations in real-world settings, offering insights into how various personal and contextual elements outside the dataset might affect participant engagement.

The variation in PR-AUC scores across the two iterations also raises questions about the adaptability of the five developed machine learning models in dynamic settings, where user behaviors and preferences may change over time. This highlights the importance of regularly updating or retraining models to reflect changes in user preferences and behaviors.

To further enhance these models, expanding the feature set to capture more detailed aspects of user engagement, such as completion rates of key intervention tasks and participation in social activities, could provide deeper insights. Additionally, incorporating study completion indicators and analyzing engagement patterns among participants who did not complete the study may reveal critical factors related to dropout. Integrating external data sources, such as user surveys and digital phenotyping from mobile device sensors, could further refine the models and enhance their applicability across diverse contexts.

Finally, the real-world utility of model interpretability is vital. While SHAP provides model insights, translating these into practical strategies is essential for boosting user engagement. The outcomes of this analysis suggest exploring enhancements in daily tasks, lesson completion, and login frequency for future eSano iterations. Refining the structure and presentation of these elements, coupled with strategies such as gamification for regular logins and targeted reminders, could deepen user involvement.

## Limitations

Reflecting on the findings of this study, several limitations are evident that merit consideration. A primary limitation is the relatively small sample size of 233 participants. While this number provided sufficient data for initial model training and validation, a larger sample would likely offer a more reliable and generalizable understanding of user engagement patterns. Another key limitation is the lack of real-time application of the machine learning models. Applying models in a real-time environment could present different challenges and opportunities, such as responding to dynamic changes in user behavior. Additionally, the study focused on a specific set of machine learning models, potentially overlooking insights that could be gained from more advanced or novel techniques like deep learning or ensemble methods. Moreover, the models were validated within the same application context from which the data was drawn, lacking external validation in different applications or contexts that would strengthen their generalizability. A further limitation is the high correlation among primary behavioral variables. This correlation is expected due to the inherent relationships between behaviors such as logging frequency, time spent on lessons, and activity completion. These also overlap with the variable ‘total_minutes’, which may have limited the depth of insights into more nuanced user behaviors. Additionally, the study focused on broader, more easily quantifiable metrics, and did not include more granular behavioral data, such as the quality of submitted answer sheets or daily diaries, due to a combination of practical, ethical, and resource considerations. Future studies should consider incorporating more diverse and uncorrelated variables, as well as detailed engagement indicators, potentially including anonymized quality ratings by clinicians, provided that the necessary ethical and data security approvals are obtained.

## Conclusion

In conclusion, this study’s exploration of machine learning models in an eHealth context has highlighted key aspects of user engagement. The significant role of ‘total_minutes’ in the first iteration, using data from the first four weeks, highlights its importance as a primary engagement indicator for predicting later weeks. However, the second iteration, which excluded ‘total_minutes’, revealed the relevance of other engagement metrics such as ‘number_intervention_answersheets’ and ‘logins_first_4_weeks’, suggesting a nuanced approach to user interactions. The variation in precision-recall AUC scores across models and iterations might reflect not just feature selection impact but also potential indistinct patterns in the data or limitations in the dataset’s size and scope. These findings are important for application and intervention developers as well as marketers, suggesting a focus on both the quantity and quality of user interactions for enhancing engagement.

## Data Availability

The datasets generated during and/or analyzed during the current study are not publicly available due to necessary data security measures, which restrict access to authorized personnel only. This ensures the protection and confidentiality of the data. Access to specific parts of these datasets can be granted under stringent conditions, subject to a rigorous approval process. Interested researchers should contact the corresponding author to request access, which will be considered on a case-by-case basis.
